# Brazil’s scientists scramble to solve the Zika puzzle

**DOI:** 10.2471/BLT.16.030316

**Published:** 2016-03-01

## Abstract

The World Health Organization has declared the recent leap in the number of microcephaly cases and their suspected association with Zika virus a public health emergency of international concern. Ana Bispo tells Andréia Azevedo Soares why Brazil should have some scientific answers in coming months.

Q: When did you first become aware of this virus?

A: I first heard of Zika at a meeting of an international network of Latin American and Iberian laboratories called ViroRed in November 2014 in Uruguay. My lab is a member of this network. Some colleagues from Europe, who also attended the meeting, were concerned about an outbreak of Zika virus in French Polynesia in 2013. They described how they had genetically sequenced Zika virus and gave participants samples of the viral ribonucleic acid (RNA), which can be used to detect the virus using a technique known as reverse transcription polymerase chain reaction or RT–PCR.

Q: Could you tell us some of the history of Zika virus in Brazil?

A: In November and December 2014, physicians from different states of the north-east region observed an outbreak of dengue-like illness and in April 2015 researchers from the Federal University of Bahia confirmed that Zika virus was the cause. Since then, 21 states have reported cases of Zika virus in Brazil. In Rio de Janeiro, the first cases were confirmed in May 2015. So Zika virus arrived in Brazil and found a very susceptible population because people had not been exposed to it before and because the main vector, the *Aedes aegypti* mosquito, was widespread. In Brazil an increase in microcephaly cases was observed in Pernambuco state, in the north-eastern part of the country, in suspected association with Zika virus. In French Polynesia cases of neurological damage and Guillain-Barré syndrome were reported. But Brazil was the first place where such a steep increase in microcephaly in babies had been observed and this increase appeared to be associated with Zika virus: almost 4000 cases of microcephaly were registered in Brazil between August and December 2015.

“We are following about 300 pregnant women."

*Q: Is your institution conducting case*–*control studies to solve the mystery of whether Zika virus is indeed a cause of microcephaly?*

A: Yes, Fiocruz in Rio de Janeiro has started a case–control study with pregnant women mainly focused on microcephaly with Zika virus. Overall, we are following about 300 pregnant women. A similar case–control study is also being conducted by colleagues at the Aggeu Magalhães Research Center, a branch of Fiocruz in Pernambuco state, where an exceptionally high number of microcephaly cases was reported last year.

Q: Can you tell us more about your lab’s work on the suspected association between microcephaly and Zika virus?

A: The first important finding was described by our team in November 2015, when we detected Zika virus genome in the amniotic fluid of two pregnant women from Paraíba state. Both women had reported a medical condition consistent with Zika virus infection. The ultrasound images of their fetuses were normal until a month after their probable infection with Zika virus, when the physician who examined them saw the first micro-calcifications of the brain. The physician confirmed that both fetuses had microcephaly and offered the women amniocentesis tests to rule out other genetic problems. Aware of the growing number of microcephaly cases, the physician called us to check whether we could detect Zika virus in their amniotic fluid. The results were positive in both cases. Our findings came about a week after the Brazilian government declared a health emergency due to the increase in microcephaly cases and so these new data were very important for guiding the investigations. Some days after we announced the amniotic fluid evidence, scientists in the state of Pará found Zika virus in samples from a baby with microcephaly that had died after birth and, recently, my team detected Zika virus in the placenta, liver, lung, umbilical cord and brain of a fetus that had been spontaneously aborted.

Q: When will it be possible to confirm whether Zika virus infection is a cause of microcephaly?

A: According to the data we have been collecting – and the new babies that will be born in the meantime – we may have concrete results in June or July.

Q: Could something else have caused the leap in the number of cases of microcephaly?

A: I doubt it. The majority of women who have delivered babies with microcephaly report that they have had a medical condition consistent with Zika, especially during the first trimester. It is hard to establish a causal link between Zika and microcephaly, because it is difficult to detect the virus when the baby is born. The Ministry of Health recommends that samples of umbilical-cord blood, placenta and urine should be collected. We do not have a test that is serologically specific enough to detect Zika virus antibodies in a baby with microcephaly. It’s the same with Guillain-Barré syndrome: when the symptoms appear (usually 12 to 15 days after Zika virus infection) patients are no longer in the acute phase of Zika disease. But we still think that Zika is responsible because when you compare current numbers of Guillain-Barré cases with those before Zika arrived in Brazil, the figures are much greater.

Q: Could the recent leap in microcephaly be in any way attributed to heightened awareness and increased surveillance?

A: You can always argue that health professionals are much more aware of such birth defects and more vigilant in terms of identifying them and, of course, we have better diagnostics in general. But, still, this is a very large increase, so it is puzzling. That’s why more research is needed to shed light on these mysteries.

“It will be easier to develop a vaccine for Zika than dengue.”

Q: Brazilian scientists are trying to develop a vaccine for Zika. What are the main challenges?

A: There are three groups of scientists in Brazil that are trying to develop a Zika vaccine. One from Bio-Manguinhos, which is part of Fiocruz, is working with GlaxoSmithKline to develop a dengue vaccine that can be used as a model for a Zika vaccine. Another group based in São Paulo, from the Butantan Institute, has started to work on a Zika vaccine with the US National Institutes of Health. And a group at the Evandro Chagas Institute is collaborating with the University of Texas Medical Branch. It will be easier to develop a vaccine for Zika than dengue because Zika has only one serotype while dengue has four. In addition, only two Zika lineages, an Asian and an African one, are described in the scientific literature.

Q: Fiocruz has just announced the creation of a rapid test to diagnose Zika, dengue and chikungunya simultaneously. Why is it innovative?

A: This is a ready-to-use multiplex PCR test that allows you to detect three viruses: dengue, chikungunya or Zika. The technicians only need to add the extracted RNA sample to the device containing three different reagents. It is called the Nucleic Acid Test for Dengue, Chikungunya and Zika viruses and is in the final phase of validation. We are hoping to complete the validation process by the end of this month. It is expected to cost about US$ 20 per test kit, including RNA extraction. The test has great potential for improving epidemiological vigilance because it shows which virus is circulating at a specific time in a given area. The test could also be particularly useful in the first days of infection, when the symptoms of Zika, chikungunya and dengue are so similar that it is difficult for doctors to distinguish between dengue and Zika, for example. The kits are also user-friendly, so they can be used by a technician with basic knowledge in real time PCR. If the final validation goes well, about 500 000 test kits could be available in the country by the end of the year. I proposed the test and my colleagues at Fiocruz’s Institute of Molecular Biology in Paraná state developed it.

Q: Although the virus is normally spread by mosquitoes, there have been reports of possible spread through sex. How strong is the evidence?

A: There is a report in the scientific literature of two cases of Zika that were sexually transmitted. Recently my team detected Zika virus in semen and urine from a patient with Zika virus infection 16 days after the symptoms of the disease appeared. Additional studies are being conducted to verify whether Zika virus can be sexually transmitted. It is difficult to collect samples of semen and vaginal fluids, but we must pursue this line of investigation. We must also look at possible infection with Zika virus via breast milk. Nevertheless, mothers are encouraged to continue breastfeeding their newborns as evidence suggests that the benefits outweigh any theoretical risks of infection.

Q: How will the Zika outbreak evolve in the coming months ahead of the Olympic Games that are scheduled to take place in Brazil in August?

A: We foresee the same scenario that we had with dengue during the 2014 World Cup, which also took place in the winter. Mosquitoes that hibernate need warm weather to become active: the *Aedes* shuts down for winter and so we did not have a big problem with dengue during the World Cup. The measures to control the vector will be as stringent as they were during the World Cup. There was huge investment in vector control, especially around football stadiums and other mass-gathering areas. The government is working hard on prevention so that visitors to Brazil – apart from pregnant women – need not worry.

